# Microwave-assisted polystyrene sulfonate-catalyzed synthesis of novel pyrroles

**DOI:** 10.1186/2191-2858-2-24

**Published:** 2012-06-22

**Authors:** Rosario Astrid Vargas Cárdenas, Blanca Olinda Quintanilla Leal, Ashwini Reddy, Debasish Bandyopadhyay, Bimal K Banik

**Affiliations:** 1 Department of Chemistry, The University of Texas-Pan American, 1201 West University Drive, Edinburg, TX, 78539, USA

**Keywords:** Pyrrole, Polystyrene sulfonate, Microwave, 2,5-dimethoxytetrahydrofuran

## Abstract

**Background:**

Pyrroles are widely distributed in nature and important biologically active molecules. The reaction of amines with 2,5-dimethoxytetrahydrofuran is a promising pathway for the synthesis of pharmacologically active pyrroles under microwave irradiation.

**Results:**

Microwave-induced polystyrenesulfonate-catalyzed synthesis of pyrroles from amines and 2,5-diemthoxytetrahydrofuran has been accomplished with excellent yield. This method produces pyrroles with polyaromatic amines.

**Conclusion:**

The present procedure for the synthesis of *N*-aromatic substituted pyrroles will find useful application in the area of pharmacologically active molecules.

## Background

**Figure 1:**
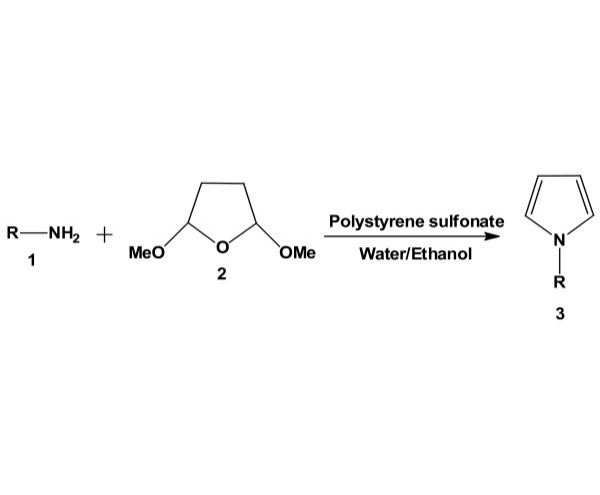
**Polystyrene sulfonate-catalyzed simple synthesis of *N*-substituted pyrroles.**

Pyrrole is one of the most significant heterocyclic structural scaffold present in a large number of biologically active molecules [1,2] with a wide range of applications in medicinal chemistry [3]. Besides its pharmacological activity, pyrrole derivatives play a crucial role in material science [4]. The traditional methods rely on the cyclization of amines with ketones or diketones discovered by Knorr and Paal in 1880 s [5,6]. Since then, a number of publications have appeared on this reaction, but still, this method is under active investigation because of its simplicity. Recently, a number of methods and catalysts have been reported, e.g., supercritical carbon dioxide [7], silver-salts promoted three component reaction [8], manganese(III)-catalyzed [3 + 2] annulation [9], rhodium(III)-catalyzed bond functionalization [10], palladium-induced three component reaction [11], gold(I)-catalyzed amino-Claisen rearrangement [12] and zinc chloride [13]. In contrast, many of these reported methods involve the use of expensive reagents, hazardous solvents, longer reaction times and tedious workup procedure. Therefore, it is desirable to develop an efficient and practical method for the synthesis of pyrrole derivatives. We have also reported several procedures in this area [14-19]. We report herein a simple microwave-assisted method for the synthesis of *N*-arylpyrroles using an aqueous solution of polystyrene sulfonate. Synthesis of pyrroles in aqueous solution is promising and demanding because of eco-friendly nature of the procedure (Figure 1).

**Figure 2:**
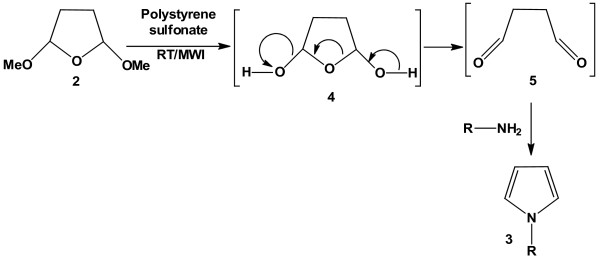
Polystyrene sulfonate- catalyzed synthesis of pyrroles: plausible mechanism of the reaction.

Although the mechanism of this reaction is not investigated, we believe compound 2 (Figure 1) produces a diketo compound in the media due to acid-induced cleavage reaction. The intermediate diketo compound then undergoes reaction with aromatic amines through nucleophilic and dehydration pathways (Figure 2). It is interesting to note that dehydration occurs in the presence of aqueous acid. Pyrroles are sensitive under strong acidic conditions, and isolation of these types of products from acidic solution may prove to be problematic. However, this problem can be overcome by simply basifying the reaction mixture prior to extraction. Higher temperature (60°C) and high power microwave radiation (300 W) accelerate the reaction significantly (Table 1). The reaction proceeds well in ethanol, THF and acetonitrile.

**Table 1:** **Polystyrene sulfonate-catalyzed simple synthesis of *N*-substituted pyrroles under microwave irradiation (300 W, 60°C) following Figure 1**

**Entry**	**Amine**	**Product**	**Time (min)**	**Yield (%)**^**a**^
1			15	96
2			20	89
3			5	90
4			25	96
5			35	94
6			50	91
7			45	93

^a^Isolated yield.

## Methods

FT-IR spectra were registered on a Bruker IFS 55 Equinox FTIR spectrophotometer (Bruker Corporation, Billerica, MA, USA) as KBr discs.^1^ H-NMR (600 MHz) and^13^ C-NMR (150 MHz) spectra were obtained at room temperature with Bruker-600 equipment (Bruker Corporation) using TMS as internal standard and CDCl_3_ as solvent. Analytical grade chemicals (Sigma-Aldrich Corporation, St. Louis, MO, USA) were used throughout the project. Deionized water was used for the preparation of all aqueous solutions.

## Results and discussion

In continuation of our research on environmentally benign reaction and biological evaluation of various polyaromatic compounds as novel anticancer agents [20-26], we have investigated reaction between aromatic amines (1) with 2,5-diemthoxytetrahydrofuran (2) using aqueous polystyrene sulfonate. After various experimentations, we have identified that this method works well under microwave-induced reaction conditions. Monocyclic, bicyclic, tricyclic and tetracyclic aromatic amines are used, and *N*-aryl pyrroles are produced in good yields (Figure 1). At the beginning of the procedure, 2,5-diemthoxytetrahydrofuran (2), the amine (1) and aqueous solution of polystyrene sulfonate were mixed in ethanol. The mixture was then stirred at room temperature, and the progress of the reaction was monitored through TLC every after 30 min. After completion of the reaction, the reaction mixture was basified with aqueous-saturated sodium bicarbonate solution and extracted with dichloromethane. The organic part was then washed with brine, dried with sodium sulfate and evaporated. The yields of the products are shown in Table 2. The multi-cyclic aromatic amines needed longer reaction time compared to the amines which are more basic and superior nucleophilic reagents.

**Table 2:** **Polystyrene sulfonate-catalyzed simple synthesis of *N*-substituted pyrroles at room temperature following Figure 1**

**Entry**	**Amine**	**Product**	**Time (h)**	**Yield (%)**^**a**^
1			9	91
2			11	85
3			8	87
4			17	90
5			21	88
6			26	92
7			23	89

^a^Isolated yield.

## Experimental

### General procedure for the synthesis of pyrroles (3)

Amine (1.0 mmol), 2, 5-dimethoxytetrahydrofuran (1.2 mmol) and polystyrene sulfonate (18 wt% solution in water) in ethanol/water (1:1) mixture was stirred at room temperature, and the progress of the reaction was monitored by TLC every 30 min. After completion of the reaction (Table 2), the reaction mixture was basified with saturated aqueous sodium bicarbonate solution and extracted with dichloromethane. The organic layer was then washed with brine, dried with sodium sulfate and evaporated to isolate the pure product.

Alternatively, amine (1.0 mmol), 2, 5-dimethoxytetrahydrofuran (1.2 mmol) and polystyrene sulfonate (18 wt% solution in water) in ethanol/water (1:1) were irradiated in an automated microwave oven (CEM Corporation, Matthews City, NC, USA). The reaction was monitored by TLC every 5 min. Depending upon the nature of the amines, the reaction was completed in different time. The result of the procedure was shown in Table 1. All the products have demonstrated satisfactory spectral and mp data with our reported compounds [16].

## Conclusions

A new and simple method for the synthesis of *N*-substituted pyrroles in aqueous solution has been investigated with success. Based on our previous studies in this series, the compounds as reported herein may demonstrate anticancer activities.

## Competing interests

The authors declare that they have no competing interests.

## Authors’ contributions

RAVC and BOQL performed the reactions with the help of AR and DB. BKB is the PI. All authors read and approved the final manuscript.
